# Nature-Inspired Polymerization of Quercetin to Produce Antioxidant Nanoparticles with Controlled Size and Skin Tone-Matching Colors

**DOI:** 10.3390/molecules24213815

**Published:** 2019-10-23

**Authors:** Suhair Sunoqrot, Eveen Al-Shalabi, Lina Hasan Ibrahim, Hiba Zalloum

**Affiliations:** 1 Department of Pharmacy, Faculty of Pharmacy, Al-Zaytoonah University of Jordan, Amman 11733, Jordan; 2 Hamdi Mango Center for Scientific Research, University of Jordan, Amman 11942, Jordan

**Keywords:** quercetin, antioxidant nanoparticles, oxidative self-polymerization, size control, green chemistry

## Abstract

Plant polyphenols have received considerable attention in recent years due to their ability to undergo oxidation-triggered self-polymerization, forming biocompatible versatile coatings and templated nanoparticles (NPs) that can be leveraged for a variety of biomedical applications. Here we show for the first time that untemplated NPs can be conveniently synthesized from the abundant plant polyphenol quercetin (QCT) simply by incubation with an oxidizing agent in a universal organic solvent, followed by self-assembly upon gradual addition of water. The process yielded NPs of around 180–200 nm in size with a range of colors that resembled light to medium-brown skin tones. The NPs were characterized by UV-Vis, FT-IR, and ^1^H-NMR spectroscopy and by dynamic light scattering and transmission electron microscopy to understand their physicochemical properties. Antioxidant and cell viability assays were also conducted to demonstrate the NPs’ free-radical scavenging activity and biocompatibility, altogether providing valuable insights into the structure and function of this emerging class of nanomaterials to guide future biomedical applications.

## 1. Introduction

Plant polyphenols have emerged as an attractive source of biocompatible and versatile coating precursors for a wide range of substrates, enabled by their ability to undergo oxidation-triggered self-polymerization similar to mussel-inspired polydopamine (PD) formation [1,2]. The same oxidative coupling mechanism has led to the development of nanoparticles (NPs) from polyphenols such as dopamine, tannic acid, and green tea catechins [3,4,5,6,7]. Of these, PD NPs are perhaps the most widely studied, with applications ranging from photothermal therapy, theranostics, drug delivery, and as UV-protective synthetic melanin-mimics [8,9,10,11,12,13,14]. PD NPs have been synthesized through a variety of approaches with the ability to control the particle size from tens to a few hundred nanometers depending on reaction conditions such as solvent composition, pH, temperature, and the oxidizing agent used [15]. An appealing feature of PD and similar polyphenol NPs is their chemical versatility, which facilitates the incorporation of various ligands and cargo for effective targeting, imaging, and therapeutic applications [16,17,18]. 

Recently, we reported on the formation of untemplated NPs from the ubiquitous plant polyphenol quercetin (QCT) [19]. QCT, which is the major representative of the flavonol family, formed NPs ~30 nm in diameter upon incubation in an aqueous buffer at pH 9. The NPs were later functionalized with amine-terminated methoxy poly (ethylene glycol) as a model surface ligand and doxorubicin as anticancer drug to demonstrate their versatility and ease of functionalization. Due to the poor aqueous solubility of QCT, the NP growth solution contained a significant amount of an organic co-solvent (25% dimethyl sulfoxide (DMSO)). Limited aqueous solubility may constitute a hurdle toward the widespread application of most plant polyphenols as NP precursors. In addition, the prototype QCT NPs may be considered too small, leading to nonspecific uptake for in vivo drug delivery applications, and there has been no attempt at controlling their particle size.

For these reasons, we devised an alternate route for QCT NP synthesis where oxidative polymerization occurs in a universal solvent such as DMSO with the aid of an oxidizing agent such as sodium metaperiodate (NaIO_4_), leading to the formation of QCT oligomers. We hypothesized that these oligomers can be further assembled into larger NPs by non-covalent self-assembly upon solvent exchange with water through dialysis in a process similar to polymeric NP self-assembly. The new synthetic route yielded size-controlled NPs with a range of colors that matched different skin tones. We characterized the NPs by various techniques to better understand their physicochemical properties. The potential for their use in biomedical applications was also evaluated by conducting antioxidant and cell viability assays.

## 2. Results

### 2.1. Synthesis and Characterization of QCT NPs

**Figure 1:**
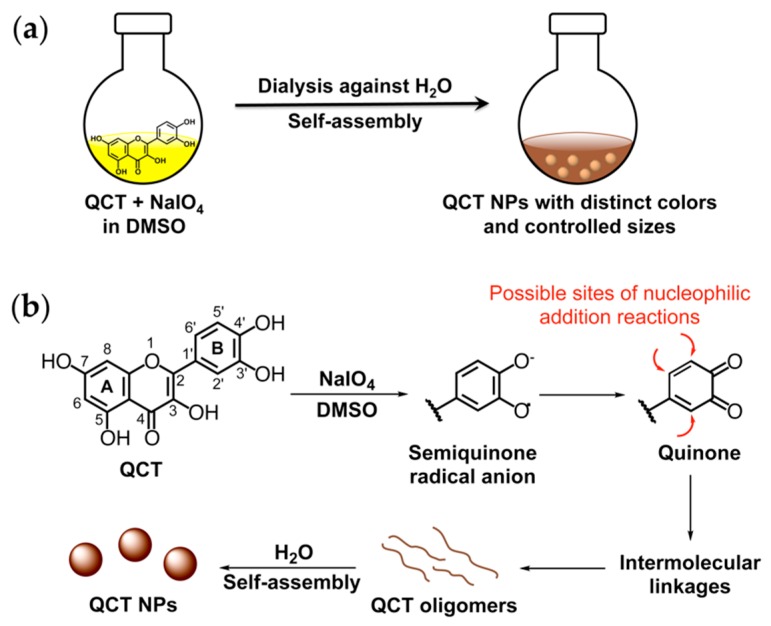
(**a**) Schematic overview of quercetin (QCT) nanoparticle (NP) synthesis; (**b**) A proposed mechanism for NP formation.

A schematic overview of QCT NP synthesis and a proposed mechanism for NP formation are displayed in Figure 1. In the presence of NaIO_4_ as oxidant, QCT undergoes a cascade of reactions leading to the formation of semiquinone radicals and reactive quinones, which render ring B susceptible to nucleophilic addition reactions, resulting in the formation of QCT oligomers. This oxidative coupling process has previously been investigated as a natural defense mechanism in the plant kingdom and is the cause of browning of air-exposed fruits and vegetables [20]. Nature-inspired polyphenol oxidation and subsequent coupling has been achieved by several approaches, the most common of which is incubation in mild alkaline solutions, where solution alkalinity promotes the deprotonation of catechol groups, leading to the formation of quinones [5,8,11]. Catechol oxidation has also been reported using oxidizing agents such as NaIO_4_ [9], copper (II) sulfate [6], and hydrogen peroxide [12]. All of these reports described polyphenol polymerization in aqueous media and mainly utilized water soluble polyphenols as NP or coating precursors. Only one study described a nonaqueous PD coating process conducted in protic organic solvents such as methanol and ethanol with the aid of piperidine [21]. To the best of our knowledge, polyphenol NP synthesis via oxidative self-polymerization has not been previously attempted in 100% aprotic organic solvents.

**Figure 2:**
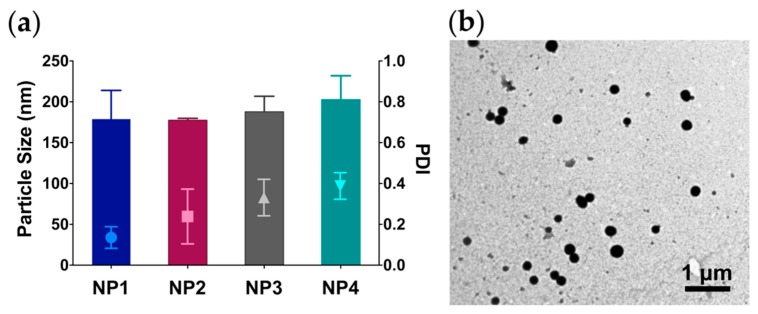
(**a**) Particle size and polydispersity indices (PDI) of QCT NPs synthesized at a QCT:NaIO_4_ ratio of 1:1, 1:2, 1:3, and 1:4 (NP1, NP2, NP3, and NP4, respectively); (**b**) Transmission electron microscopy (TEM) image of NP1.

As shown in Figure 2a and Table S1, QCT oxidation by NaIO_4_ and subsequent self-assembly led to the formation of NPs ranging from 177.4 to 202.6 nm in diameter as measured by dynamic light scattering (DLS), regardless of the NaIO_4_ concentration. NPs were also characterized by a spherical morphology and high electron density as observed under transmission electron microscopy (TEM) (Figure 2b and Figure S1). Interestingly, all NPs demonstrated comparable particle size yet exhibited a color gradient from light orange to amber brown as the concentration of NaIO_4_ in the reaction medium was increased. In addition, we noticed an increase in NP polydispersity indices (PDI) as the NaIO_4_ concentration was increased, with NP1 (synthesized at a QCT:NaIO_4_ ratio of 1:1) exhibiting the lowest PDI of 0.14, compared to NP4 (synthesized at a QCT:NaIO_4_ ratio of 1:4) with an average PDI of 0.39 (*p* < 0.05). All NPs carried a partially negative surface charge with zeta potential values ranging from –5.1 to –1.4 mV, attributed to the high electron density of the aromatic groups in QCT (Table S1). Additionally, the NPs were found to contain minute amounts of unchanged QCT as measured by HPLC (Table S2). 

**Figure 3:**
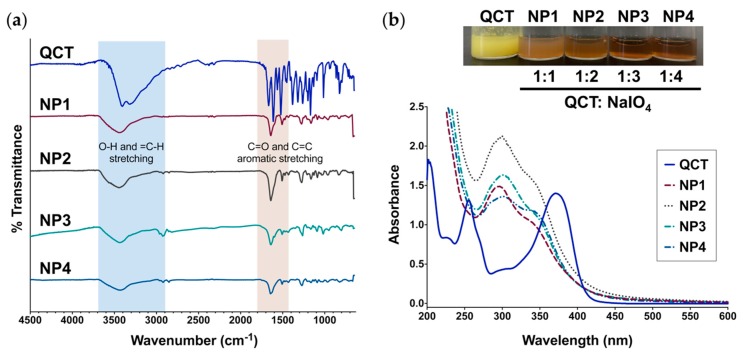
(**a**) FT-IR spectra of QCT and the NPs prepared in this study showing the characteristic bands for O-H stretching (3300–3700 cm^−1^), C=C-H stretching (2900–3000 cm^−1^), and C=O/C=C aromatic stretching (1400–1800 cm^−1^); (**b**) UV-Vis spectra of QCT and QCT NPs. Top: Aqueous dispersions of QCT NPs synthesized at a QCT:NaIO_4_ ratio of 1:1, 1:2, 1:3, and 1:4 (NP1, NP2, NP3, and NP4, respectively) next to QCT. UV-Vis revealed a shift in the characteristic absorption bands of QCT after oxidation and NP formation, accompanied by a distinct change in color.

FT-IR spectroscopy of QCT NPs exhibited characteristic –OH, C=C-H, C=O, and C=C aromatic stretching bands similar to QCT, indicating that no new functional groups were formed during NP synthesis (Figure 3a). NPs dispersed in water showed almost identical UV-Vis spectra, which, compared to QCT, were all characterized by a blue shift in band I from 374 nm to ~350 nm and a red shift in band II from 256 nm (QCT) to ~300 nm at all ratios of NaIO_4_ (Figure 3b). When employing NaOMe and AlCl_3_ as shift reagents [19,22,23], all NPs displayed similar spectral changes (Table S3 and Figure S2). Addition of NaOMe to QCT and QCT NPs caused a red shift in band I from 374 to 426–430 nm, confirming the presence of a free –OH at C3 in all samples. Moreover, the appearance of a new peak at 329 nm indicated the presence of a free –OH group at C7. Similar results were obtained with NP1–NP4 (Table S3). Upon the addition of AlCl_3_, we noted a red shift in band I from 374 to 459 nm for QCT, attributed to the formation of a coordination complex with Al^+3^ at C3′-OH and C4′-OH, as well as C4 carbonyl and C3-OH and/or C5-OH (Figure S2). This shift was partially reversed upon adding HCl to the QCT solution due to the dissociation of Al^+3^ at C3′-OH and C4′-OH only. In the case of QCT NPs, addition of AlCl_3_ also resulted in a red shift for band I from 350 to 430 nm. Because the shift was not affected by the addition of HCl, the complexation with AlCl_3_ most likely occurred between the C4 carbonyl and C3-OH and/or C5-OH only, which could not be dissociated with the addition of HCl. This verifies the presence of free –OH groups at C3 and C5 in all NP samples and the absence of 3′,4′-dihydroxyl groups on ring B, signifying that at least one of them had been oxidized (Figure S2). 

The ^1^H-NMR spectrum of QCT (Figure S3) showed a pair of doublets at δ 6.20 and 6.41 ppm, corresponding to ring A protons H-6 and H-8, respectively. Ring B protons appeared at δ 6.91, 7.54, and 7.69 ppm for H-5′, H-6′, and H-2′, respectively. The downfield singlets at δ 9.32, 9.36, 9.60, 10.79, and 12.50 ppm refer to hydroxyl groups at C7, C4′, C3′, C3, and C5, respectively. On the other hand, ^1^H-NMR spectroscopy of QCT NPs (Figures S4–S7) revealed significant broadening in singlet and doublet peaks corresponding to aromatic and –OH protons of QCT. Peak broadening is a characteristic feature of polymeric materials [24] and is in agreement with the formation of QCT oligomers having multiple protons with overlapping chemical shift values. For example, the ^1^H-NMR spectrum for NP1 (Figure S4) showed a multiplet in the range 5.97–6.48 ppm corresponding to ring A protons H-6 and H-8. Ring B protons (H-5′, H-6′, and H-2′) also appeared as a multiplet between 6.71 and 7.33 ppm. The hydroxyl groups at C7, C4′, and C3′ appeared as a broad multiplet in the range of 8.68–9.28 ppm. A multiplet appearing between 10.75 and 11.49 ppm corresponds to the hydroxyl group at C3, and the multiplet in the range of 12.35–12.51 refers to the hydroxyl group at C5. Similar spectral changes were observed in QCT NPs synthesized at a QCT:NaIO_4_ ratio of 1:2, 1:3, and 1:4 (NP2, NP3, and NP4, respectively; Figures S5–S7), all of which strongly support the formation of oligomeric structures upon QCT oxidation.

A total phenol content assay was performed to determine the number of –OH groups remaining in QCT NPs compared to unmodified QCT. As depicted in Figure S8, NP1 was found to contain only 250.2 μg QCT equivalents/mg NP, indicating that approximately 25% of QCT’s total phenols were present. This value was significantly higher than for NP2–NP4, which were found to contain 162.9–184.5 μg QCT equivalents/mg NP (16–18% of the total phenols in QCT). The results are consistent with QCT oxidation, and strongly indicate the formation of oligo/polymeric structures containing a fewer number of phenolic –OH per weight compared to simple QCT molecules.

### 2.2. Reassembly and Labeling of QCT NPs

**Figure 4:**
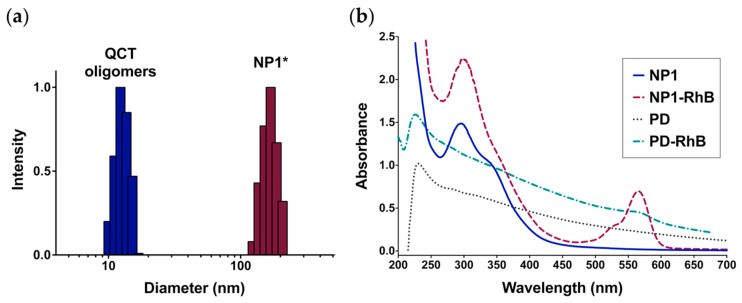
Disassembly of QCT NPs synthesized at a QCT:NaIO_4_ ratio of 1:1 (NP1) produces oligomers than can be reassembled and loaded with rhodamine B (RhB). (**a**) Intensity-weighted particle size distribution for QCT oligomers obtained by dissolving lyophilized NP1 in dimethyl sulfoxide (DMSO) compared to reassembled NP1 (NP1*), providing evidence that QCT NPs are formed by noncovalent self-assembly of smaller oligomers; (**b**) UV-Vis spectra of NP1 and RhB-labeled NP1 (NP1-RhB) showing distinct bands for QCT (270–400 nm) and RhB (500–600 nm). Conversely, RhB-labeled polydopamine NPs (PD-RhB) exhibit broad absorption across the entire UV-Vis region, which limits accurate detection of RhB.

In order to verify whether QCT NPs were formed by noncovalent self-assembly of smaller oligomers, NP1 was lyophilized, redissolved in DMSO, and then dialyzed against water, a commonly used procedure for NP formation from block copolymers. NP1 was chosen for this experiment given that it was associated with the lowest PDI among the formed NPs. Interestingly, the particle size for the DMSO solution prior to dialysis was measured to be 11.2 ± 1.0 nm (Figure 4a), which likely represents the particle size of QCT oligomers. Upon solvent exchange with water, the oligomers reassembled into larger NPs (NP1*; Figure 4a) with an average diameter of 170.0 ± 25.3 nm, which was not significantly different from freshly prepared NPs. This indicates that QCT oligomers can reversibly disassemble and reassemble, which can be conveniently employed to entrap drug payloads during NP reassembly.

We examined the possibility of labeling QCT NPs with commonly used fluorophores such as rhodamine B (RhB), which can also serve as a surrogate for small molecule drugs. The dye was not added to the NP precursors during synthesis, as the incubation with NaIO_4_ may lead to undesired oxidation of RhB. Rather, dye loading was achieved by dissolving lyophilized NP1 in DMSO with RhB, followed by repetitive dialysis against water. The process yielded NPs with an average diameter of 155.2 ± 24.0 nm. As seen in Figure 4b, the characteristic absorption band for RhB appears at 555 nm. Since QCT NPs are characterized by relatively narrow absorption bands between 275–400 nm, this would allow the accurate detection and quantitation of chromophores absorbing at 400 nm or higher such as RhB, making them useful colloidal carriers for imaging applications. Conversely, PD NPs are characterized by a broad absorption band in the UV-Vis region (Figure 4b), which interferes with the accurate determination of molecules absorbing across the UV-Vis spectrum and can even result in quenching of fluorescence emission spectra for associated dye molecules [25].

### 2.3. Antioxidant Activity of QCT NPs

**Figure 5:**
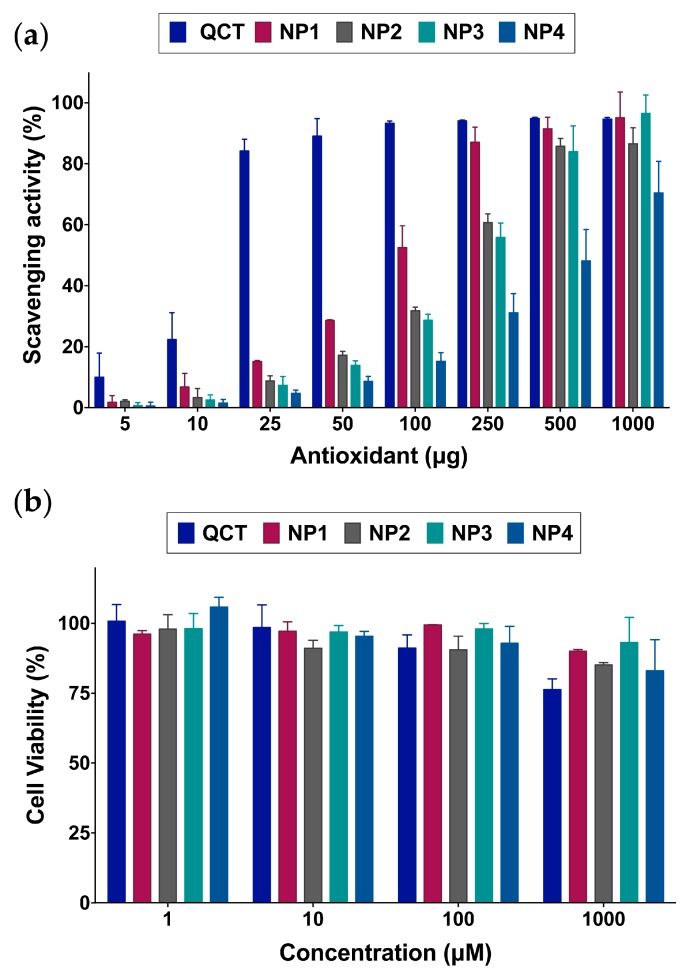
(**a**) 2,2-diphenyl-1-picrylhydrazyl (DPPH) radical scavenging activity of the NPs compared to QCT indicating a gradual decrease in antioxidant activity as the ratio of NaIO_4_ is increased from 1 to 4, with NP1 exhibiting the greatest activity after QCT; (**b**) Viability of human dermal fibroblasts treated with various concentrations of the NPs up to 72 h. QCT NPs are well tolerated by the cells up to 1000 μM. QCT started to exhibit a decrease in cell viability at 1000 μM, attributed to its inhibitory effect on fibroblast differentiation and proliferation.

2,2-diphenyl-1-picrylhydrazyl (DPPH) radical scavenging assays were conducted to determine the effect of the NP synthesis process on QCT’s antioxidant activity. As can be seen in Figure 5a, QCT is a potent antioxidant with as little as 15.1 μg resulting in 50% scavenging activity. It was evident that NP synthesis by reacting with NaIO_4_ led to a decrease in antioxidant activity, consistent with previous findings [5,19]. Nonetheless, QCT NPs still maintained free-radical scavenging activity in descending order of the QCT:NaIO_4_ ratio used in the synthesis, with 89.9, 162.7, 243.4, and 1387.0 μg of NP1, NP2, NP3, and NP4, respectively, required to achieve 50% scavenging activity. In order to verify whether the reduction in antioxidant activity resulted from kinetic differences in DPPH reactivity toward free QCT compared to the NPs, the reduction in DPPH radical absorbance at 517 was monitored during the first 10 min of the reaction. As shown in Figure S9, QCT exhibited the fastest decline in absorbance, followed by NP1, and then NP2–NP4. The slower reaction kinetics observed in QCT NPs compared to free QCT may in part be attributed to decreased accessibility of DPPH to the NPs. However, all NPs demonstrated comparable particle sizes yet different reaction kinetics, particularly between NP1 and NP2–NP4. Thus, it is more likely that the reduction in antioxidant activity was mainly attributed to the decrease in the number of available oxidizable –OH groups as the NaIO_4_ concentration increased during NP synthesis. This is supported by the fact that almost all QCT was consumed during NP synthesis as measured by HPLC (Table S2) and the significant reduction in total phenol content compared to unmodified QCT (Figure S8). 

### 2.4. Biocompatibility of QCT NPs

We have previously shown that QCT loses its anticancer activity upon polymerization to form NPs, and that QCT NPs are nontoxic to cancer cells [19]. In this study, cell viability assays in human dermal fibroblasts were used to further evaluate the biocompatibility of the synthesized NPs. As depicted in Figure 5b, QCT exhibited low toxicity up to 100 μM after 72 h incubation. At 1000 μM, QCT began to show a decrease in cell viability, reaching 76.2%. The reduction in cell viability observed with QCT-treated cells may be attributed to its inhibitory action on fibroblasts proliferation by increasing the expression of surface αV integrin and decreasing β1 integrin [26]. On the other hand, NP1–NP4 all displayed minor effects on cell viability across the concentration range tested, indicating their biocompatibility. This also confirms that most of the QCT initially used to synthesize the NPs was consumed during NP formation, rendering the NPs pharmacologically inert, which supports their use as vehicles for imaging and drug delivery applications. 

Although the existence of QCT in an oxidized state in the NPs may pose toxicological concerns due to the presence of quinones, the oligomeric nature of these oxidized moieties is expected to hinder their binding to cellular components. This is evidenced by the fact that PD NPs, which are formed by a similar oxidative coupling mechanism, have been shown to exhibit efficient free radical scavenging effects and effective reduction in intracellular oxidative stress, despite the fact that they contained quinones [27,28,29].

## 3. Materials and Methods

### 3.1. Materials

Quercetin hydrate (QCT) and potassium bromide (KBr) were obtained from Acros Organics (Geel, Belgium). Dopamine hydrochloride (DA), rhodamine B (RhB), bicine, 2,2-diphenyl-1-picrylhydrazyl (DPPH), aluminum chloride (AlCl_3_), hydrochloric acid (HCl, 10 N), and sodium methoxide (NaOMe) were obtained from Sigma-Aldrich (St. Louis, MO, USA). Dimethyl sulfoxide (DMSO) was obtained from Tedia (Fairfield, OH, USA). 3-(4,5-dimethyl-2-thiazolyl)-2,5-diphenyl-2H-tetrazolium bromide (MTT) was obtained from Promega (Madison, WI, USA). Ultrapure water (specific resistivity ~18.2 MΩ.cm at 25 °C) was prepared using a Millipore Direct-Q 5UV system (Billerica, MA, USA).

### 3.2. Synthesis of QCT NPs

QCT NPs were synthesized by oxidative self-polymerization of QCT in the presence of NaIO_4_. In a typical procedure, QCT (10 mg, 0.033 mmol) was dissolved in 1 mL DMSO containing various amounts of NaIO_4_ corresponding to 1–4 molar excess of QCT. The solution was vigorously stirred at room temperature (RT) overnight. The next day, the DMSO solution was transferred to a dialysis membrane with 3.5 kD molecular weight cutoff (MWCO; Spectrum Laboratories Inc., Rancho Dominguez, CA, USA) and dialyzed against deionized water (4 L) for one day, changing the water every hour for the first 12 h. NPs were later centrifuged at 4,000× *g* for 5 min (Hermle Z230A centrifuge, Wehingen, Germany) to remove large aggregates. NPs were either stored as an aqueous dispersion at RT or lyophilized using a FreeZone 4.5 L Benchtop Freeze Dryer (Labconco Corporation, Kansas City, MO, USA).

### 3.3. High Performance Liquid Chromatography (HPLC) Analysis

HPLC was employed to quantify the amount of QCT remaining unchanged in the synthesized NPs. Analysis was performed using a Finnigan Surveyor LC Pump Plus system (Thermo Fisher Scientific, Waltham, MA, USA) equipped with an autosampler and a photodiode array detector. Samples were eluted on a C18 UniverSil column (5 μm, 150 × 4.6 mm; Fortis Technologies Ltd., Cheshire, UK) using a mobile phase composed of 0.1% aqueous formic acid and methanol (60:40 *v*/*v*) at a flow rate of 1 mL/min, and the detection wavelength was 370 nm. QCT serial dilutions (0.16–5 μg/mL) were prepared in the mobile phase and used to construct the calibration curve. One milligram of each NP was dissolved in 1 mL of mobile phase and injected into the column. The concentration of QCT remaining in each NP was calculated based on a calibration curve of QCT standards’ peak area versus concentration. The results were expressed as % QCT content by weight.

### 3.4. Particle Size and Zeta Potential Measurements

Particle size and zeta potential of the NPs prepared in this study were measured by dynamic light scattering (DLS) using a Nicomp Nano Z3000 instrument (Particle Sizing Systems, Santa Barbara, CA, USA). Each measurement was performed in triplicate.

### 3.5. Transmission Electron Microscopy (TEM) Imaging

A drop of each NP suspended in ultrapure water was placed on 300-mesh Formvar-coated copper grids (Electron Microscopy Sciences, Hatfield, PA, USA) for 1 min. Excess liquid was blotted with filter paper. Grids were imaged using a Morgagni 268 TEM (FEI, Netherlands) at an accelerating voltage of 60 kV.

### 3.6. FT-IR, UV-Vis, and ^1^H NMR Spectroscopy

FT-IR spectra of QCT and QCT NPs were recorded using a Shimadzu IR Affinity-1 spectrometer (Kyoto, Japan), where all samples were prepared as KBr discs. UV-Vis spectroscopy was used to determine the presence of free –OH groups using NaOMe and AlCl_3_/HCl as shift reagents [19,22,23]. First, the spectra of QCT and QCT NPs (10 μg/mL in methanol) were recorded. NaOMe (three drops, 1 M in methanol) was then added to each sample and the spectra were immediately recorded. AlCl_3_ (six drops, 50 mg/mL in methanol) was added to another set of samples and the spectra were recorded. HCl (three drops, 5 M) was then added to the AlCl_3_ samples and the spectra were immediately recorded. All analyses were performed using a UV-1800 spectrophotometer (Shimadzu). ^1^H-NMR spectra were recorded in DMSO-d_6_ using a Bruker 400 MHz instrument (Billerica, MA, USA).

### 3.7. Total Phenol Content Assay

The number of phenolic –OH groups remaining in QCT NPs after synthesis was determined by the Folin–Ciocalteu method as previously described with some modification [30]. Briefly, QCT and QCT NPs were prepared as 1 mg/mL stock solutions in DMSO. QCT dilutions were prepared in the range 5–100 μg/mL, and 250 μL of each concentration were transferred to a 15-mL conical tube. Fifty microliters of each NP stock solution was diluted to 250 μL with DMSO and placed in a 15-mL conical tube. One milliliter of Folin–Ciocalteu’s phenol reagent (2 N, Sigma-Aldrich) diluted 10 times with ultrapure water was added to each conical tube, followed by the addition of 1 mL sodium bicarbonate (10% *w/v* in ultrapure water). The samples were incubated at RT in the dark for 30 min, and then the UV absorbance was measured at 765 nm using a solution composed of 1 mL phenol reagent, 1 mL sodium bicarbonate, and 250 μL DMSO as blank. QCT dilutions were used to construct a calibration curve of absorbance at 765 nm versus concentration, from which the total phenol content in QCT NPs was determined and results were expressed as μg QCT equivalents per mg NP. The experiment was performed in triplicate. 

### 3.8. Synthesis of Reassembled QCT NPs

Reassembly of QCT NPs was examined by dissolving lyophilized NPs (5 mg) in 0.5 mL DMSO and vortex mixing for 10 sec. The samples were then transferred to a dialysis membrane (3.5 kD MWCO) and dialyzed against deionized water for one day. For the synthesis of RhB-labeled NPs, lyophilized NPs (5 mg) were co-dissolved with 0.05 mg RhB in 0.5 mL DMSO prior to repetitive dialysis (3.5 kD MWCO membrane) against deionized water for three days.

### 3.9. Synthesis of Polydopamine (PD) NPs

PD NPs were synthesized as previously described with some modification [7]. Briefly, DA (12.5 mg) was dissolved in ultrapure water (44.33 mL), followed by adding bicine buffer (100 mM, 5.67 mL, pH 8.5), and the reaction was stirred gently for 24 h. The next day, the NPs were washed by ultracentrifugation (Hermle Z230A centrifuge) at 20,000× *g* and 4 °C for 40 min. The supernatant was discarded and the pellet was redispersed in ultrapure water and centrifuged again under the same conditions. Finally, the NPs were redispersed in 5 mL ultrapure water and stored at RT. RhB-labeled PD NPs (PD-RhB) were prepared by centrifuging freshly prepared NPs (20,000× *g*, 4 °C, 40 min) and redispersing the pellet in 2 mL 0.01 M NaOH containing 0.1 mg/mL RhB, followed by vigorous stirring for 24 h protected from light. Excess RhB was removed by repetitive dialysis (3.5 kD MWCO membrane) against deionized water for three days.

### 3.10. Antioxidant Activity

DPPH radical scavenging activity of QCT NPs compared to QCT was tested as previously reported [19]. For the assay, 5 mg/mL stock solutions of QCT and NP1–NP4 were prepared in ethanol. Varying amounts of each material spanning 0–1000 μg were withdrawn and diluted up to 200 μL with ethanol. The solutions were immediately added to 4 mL DPPH (0.1 mM in ethanol) and incubated at RT in the dark for 30 min. The absorbance of the solutions was then measured at 517 nm by UV-Vis (UV-1800 spectrophotometer, Shimadzu). Scavenging activity (I) was calculated according to Equation (1):

I = [1 − (A_sample_ − A_blank_)/A_DPPH_] × 100% (1)

where A_sample_ is the absorbance of each test material incubated with DPPH, A_blank_ is the background absorbance of each test material without DPPH, and A_DPPH_ is the absorbance of DPPH alone. 

DPPH radical scavenging kinetics were also evaluated by adding 100 μg of QCT and QCT NPs dissolved in 200 μL ethanol to 4 mL DPPH (0.1 mM in ethanol) and immediately measuring the UV absorbance at 517 nm at 10 sec intervals up to 10 min using the kinetics mode on a UV-1800 spectrophotometer (Shimadzu). Results were plotted as absorbance at 517 nm for each sample versus time.

### 3.11. Effect of QCT NPs on Cell Viability

Effect of QCT NPs on cell viability was evaluated in human dermal fibroblasts by means of an MTT assay. Cells were obtained from the American Type Culture Collection (ATCC, Manassas, VA, USA) and cultured in Dulbecco’s Modified Eagle’s Medium (DMEM; Euroclone, Pero, Italy) in a 37 °C, 5% CO_2_ humidified incubator. The media was supplemented with 10% heat-inactivated fetal bovine serum (FBS) (Gibco, Invitrogen, Thermo Fisher Scientific, Waltham, MA, USA), 1% of 2 mM L-glutamine (Lonza, Basel, Switzerland), 50 IU/mL penicillin (Lonza), and 50 μg/mL streptomycin (Lonza). For the experiment, cells were seeded in 96-well plates at 1.0 × 10^4^ cells per well (*n* = 4) for 24 h. The next day, media was removed and cells were treated with QCT and QCT NPs (NP1–NP4) at concentrations ranging from 0–1000 µM diluted in complete DMEM for 72 h. At the end of the incubation period, media was removed and replaced with fresh complete media. Fifteen microliters of MTT reagent (5 mg/mL) was added to each well. The plates were then incubated for 3 h, followed by addition of 100 µL of solubilization solution to each well to dissolve formazan crystals. Finally, absorbance was recorded at 540 nm using a Biotek ELx808 microplate reader (Winooski, VT, USA). Cell viability was expressed as % cell viability relative to cells treated with complete media only.

### 3.12. Statistical Analysis

Statistical analysis was performed in Graphpad Prism 6.0e using two-way analysis of variance (ANOVA) followed by Tukey’s multiple comparisons test, where *p* < 0.05 was considered statistically significant.

## 4. Conclusions

In summary, we present a novel approach to control the synthesis of organic functional NPs from QCT, an abundant water-insoluble plant polyphenol. The NPs were formed by a combination of covalent coupling of oxidized QCT molecules to form oligomers, and noncovalent self-assembly of oligomers into larger NPs. This green straightforward process was performed without requiring heat or metal catalysts, unlike previous reports involving PD and green tea catechins [4,5,31]. NPs formed at a QCT:NaIO_4_ ratio of 1:1 (NP1) exhibited the highest monodispersity and greatest free-radical scavenging activity, supporting their potential as topical UV-protective agents. QCT NPs also possess the added advantage of having cosmetically appealing colors that match light to medium-brown skin tones, unlike PD NPs, which are typically dark brown/black in color. QCT NPs may be further adapted for drug delivery applications by co-incubating preformed oligomers with the molecule of interest, which becomes entrapped during self-assembly, as demonstrated by loading of RhB. The relatively narrow UV absorption spectrum for QCT NPs allows the accurate detection of common chromophores such as RhB for imaging applications. Cell viability assays in human dermal fibroblasts revealed that the NPs were well tolerated, although a more thorough toxicological profile should still be established for QCT NPs to further support their use for biomedical applications. 

## Supplementary Materials

The following are available online at https://www.mdpi.com/1420-3049/24/21/3815/s1: Characterization of the NPs by TEM, DLS, UV-Vis, and ^1^H-NMR, and results from total phenol content and DPPH assays.
