# Supplementary material for: Nature-Inspired Polymerization of Quercetin to Produce Antioxidant Nanoparticles with Controlled Size and Skin Tone-Matching Colors
Source: Molecules. 2019 Oct 23;24(21):3815. doi: 10.3390/molecules24213815 (PMC6864733; doi:10.3390/molecules24213815)
Supplement: Supplementary file 1 [file molecules-24-03815-s001.pdf]

Supporting Information for:

Nature-inspired Polymerization of Quercetin to Produce Antioxidant Nanoparticles with Controlled Size and Skin Tone Matching Colors

Suhair Sunoqrot [1,*], Eveen Al-Shalabi [1], Lina Hasan Ibrahim [1] and Hiba Zalloum [2]

[1] Department of Pharmacy, Faculty of Pharmacy, Al-Zaytoonah University of Jordan, Amman 11733, Jordan
[2] Hamdi Mango Center for Scientific Research, University of Jordan, Amman 11942, Jordan
* Correspondence: suhair.sunoqrot@zuj.edu.jo; Tel.: +962-6-4291511

Table S1. Characterization of the NPs prepared in this study.

NP	QCT: $NaIO_4$	Particle Size (nm)	PDI	Zeta Potential (mV)
**NP1**	1:1	178.9 ± 35.1	0.14 ± 0.05	-4.6 ± 2.3
**NP2**	1:2	177.4 ± 2.4	0.24 ± 0.14	-3.0 ± 1.3
**NP3**	1:3	187.8 ± 19.0	0.34 ± 0.09	-5.1 ± 2.0
**NP4**	1:4	202.6 ± 29.3	0.39 ± 0.07	-1.4 ± 1.4

Table S2. QCT content by weight in QCT NPs measured by HPLC.

Sample	QCT content (wt%)
**NP1**	0.080
**NP2**	0.013
**NP3**	0.010
**NP4**	0.004

Table S3. Band I shifts in the UV-Vis spectra of QCT and QCT NPs upon the addition of various shift reagents.

Sample	No addition	NaOMe	$AlCl_3$	$AlCl_3$/HCl
**QCT**	374 nm	426, 329* nm	459 nm	430 nm
**NP1**	350 nm	430, 332* nm	430 nm	430 nm
**NP2**	350 nm	430, 333* nm	430 nm	430 nm
**NP3**	350 nm	430, 335* nm	430 nm	430 nm
**NP4**	348 nm	425, 336* nm	425 nm	425 nm

*New peak

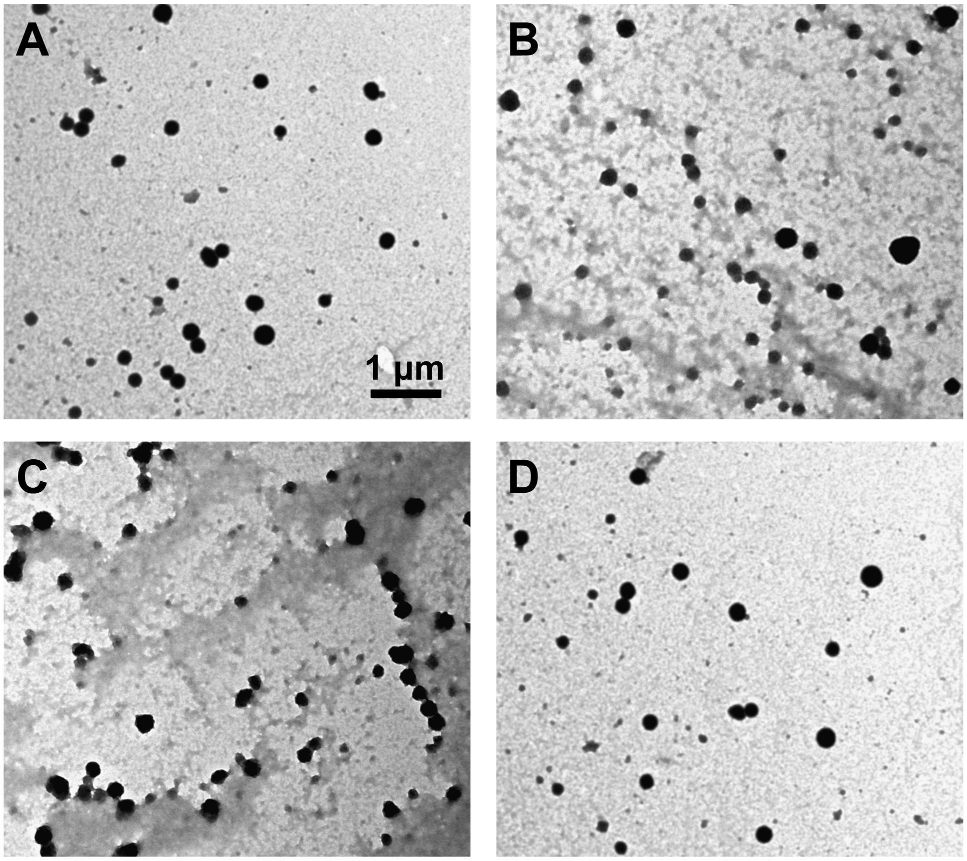

Figure S1. TEM images of (**a**) NP1, (**b**) NP2, (**c**) NP3, and (**d**) NP4.

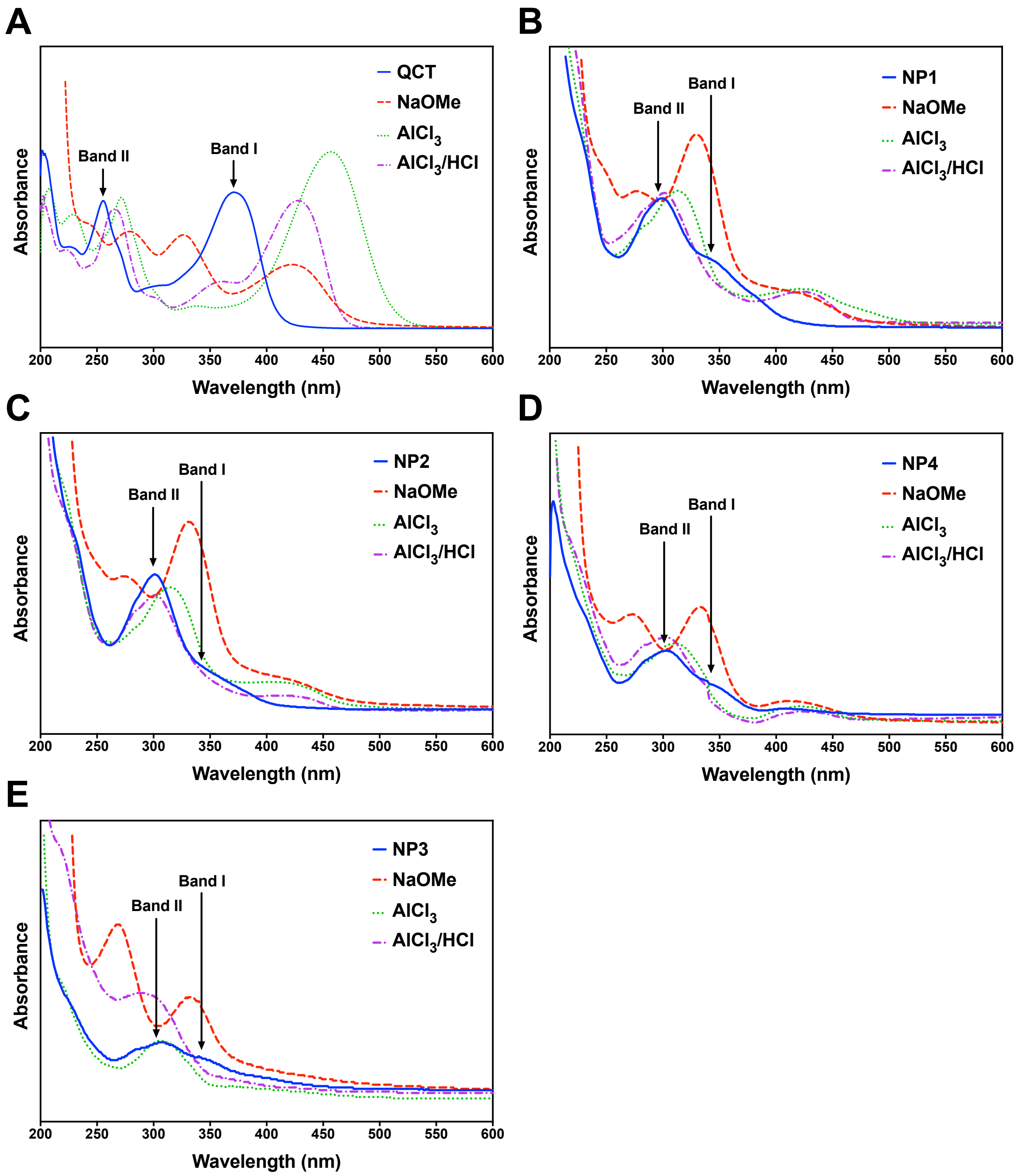
A
QCT
NaOMe
AlCl3
AlCl3/HCl
Band II
Band I
Absorbance
Wavelength (nm)
200 250 300 350 400 450 500 550 600
B
NP1
NaOMe
AlCl3
AlCl3/HCl
Band II
Band I
Absorbance
Wavelength (nm)
200 250 300 350 400 450 500 550 600
C
NP2
NaOMe
AlCl3
AlCl3/HCl
Band II
Band I
Absorbance
Wavelength (nm)
200 250 300 350 400 450 500 550 600
D
NP4
NaOMe
AlCl3
AlCl3/HCl
Band II
Band I
Absorbance
Wavelength (nm)
200 250 300 350 400 450 500 550 600
E
NP3
NaOMe
AlCl3
AlCl3/HCl
Band II
Band I
Absorbance
Wavelength (nm)
200 250 300 350 400 450 500 550 600

F

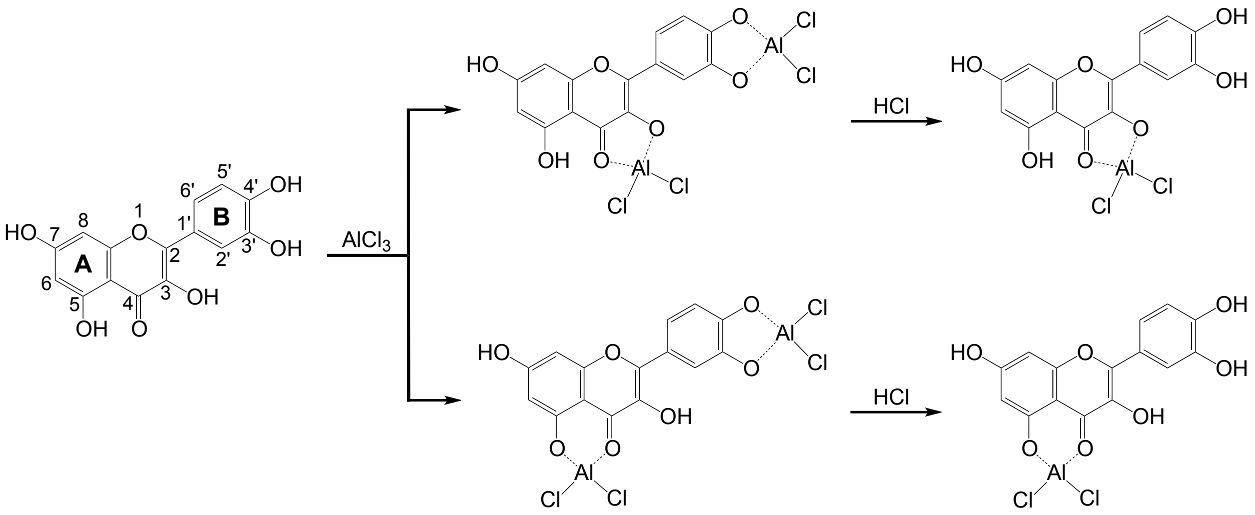

Figure S2. UV-Vis spectra of methanolic solutions of (**a**) QCT, (**b**) NP1, (**c**) NP2, (**d**) NP3, and (**e**) NP4 before and after the addition of NaOMe, $AlCl_3$, and $AlCl_3$/HCl; (**f**) Types of complexes that can form between $AlCl_3$ and QCT. Addition of NaOMe to QCT and QCT NPs causes a red shift in band I from 374 to 426 – 430 nm, confirming the presence of a free –OH at C3 in all samples. The appearance of a new peak at ~330 nm indicates the presence of a free –OH group at C7. Complexation with $AlCl_3$ causes a red shift in band I for all samples. If the 3′,4′-dihydroxyl groups are available, the complex formed with Al^{+3} at that position can be dissociated upon the addition of HCl, resulting in partial shift in band I back to a lower wavelength. If the shift in band I upon the addition of $AlCl_3$ is not affected by subsequent addition of HCl as in the case of QCT NPs, this means that the complex was formed between Al^{+3}, C4 carbonyl, and C3-OH and/or C5-OH only. This strongly indicates that at least one of the 3′,4′-dihydroxyl groups on ring B has been oxidized, becoming unavailable for complexation with $AlCl_3$.

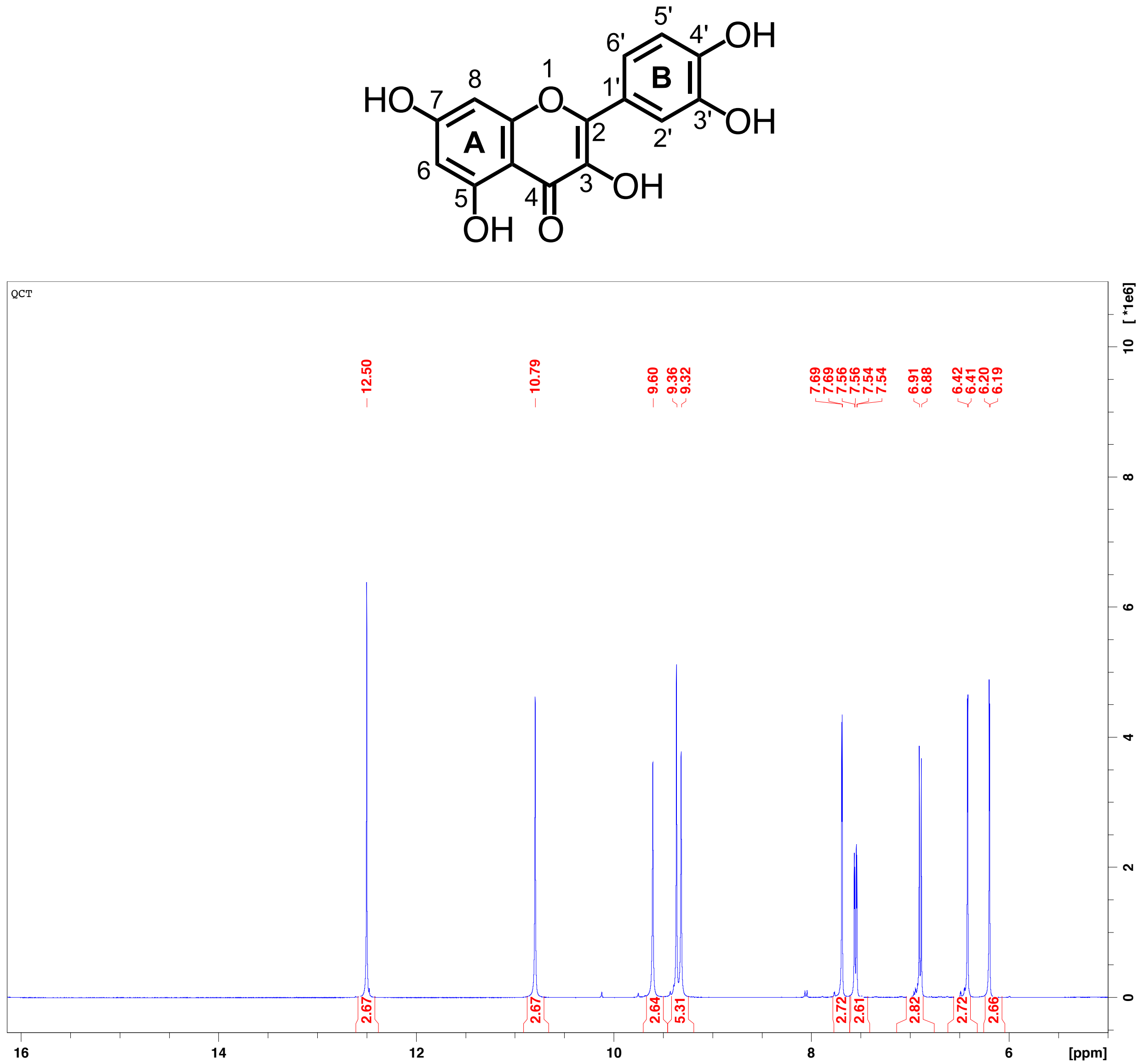

Figure S3. ^{1}H-NMR spectrum of QCT (400 MHz, DMSO-d6). δ: 6.20 (1H, d, H-6), 6.41 (1H, d, H-8), 6.91 (1H, d, H-5′), 7.54 (1H, d, H-6′), 7.69 (1H, s, H-2′); 9.32 (1H, s, C7-OH), 9.36 (1H, s, C3′-OH), 9.6 (1H, s, C4′-OH), 10.79 (1H, s, C3-OH), 12.50 (1H, s, C5-OH) ppm.

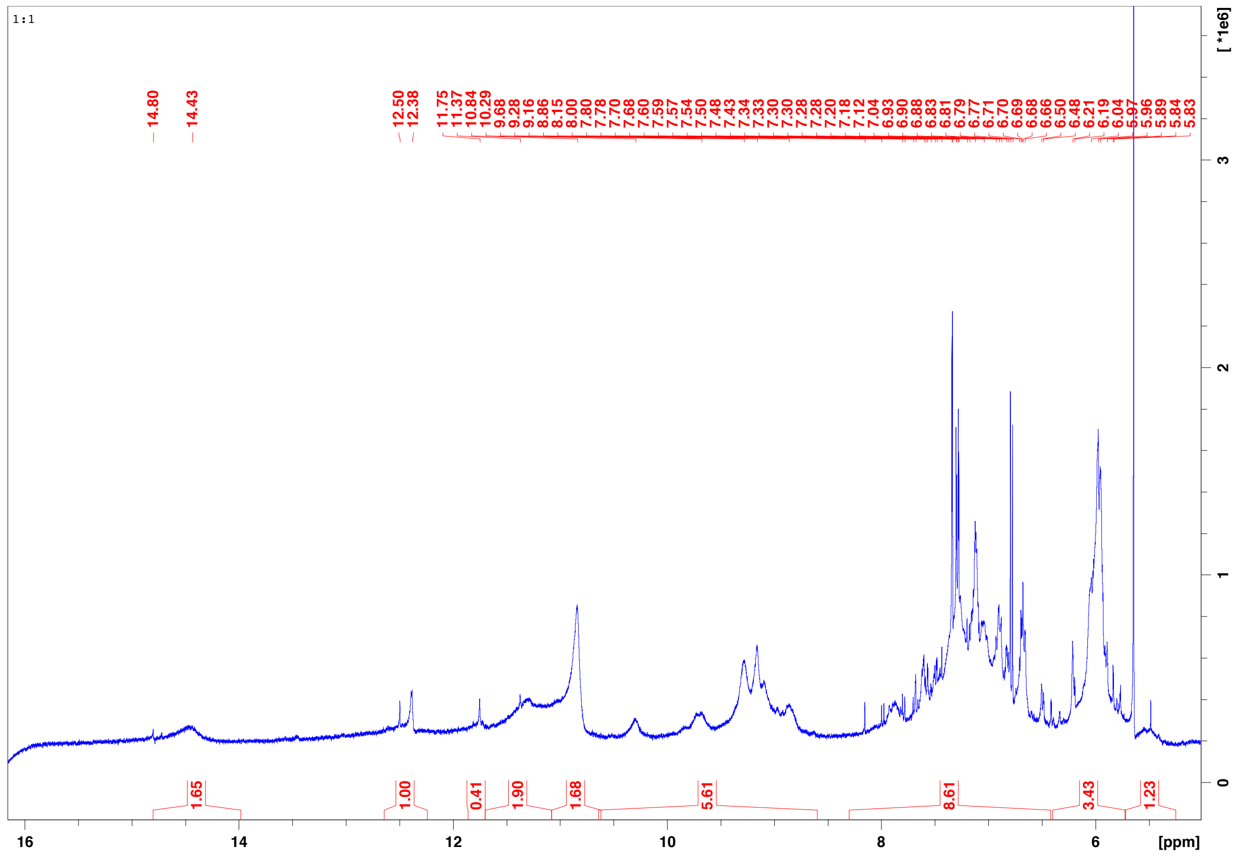

Figure S4. ^{1}H-NMR spectrum of NP1 (400 MHz, DMSO-d6). δ: 5.97 – 6.48 (Ar-H, m, H-6, H-8), 6.71 – 7.33 (Ar-H, m, H-5′, H-6′, H-2′); 8.68 – 9.28 (–OH, m, C7-OH, C4′-OH, C3′-OH), 10.75 – 11.49 (–OH, m, C3-OH), 12.35 – 12.51 (–OH, m, C5-OH) ppm.

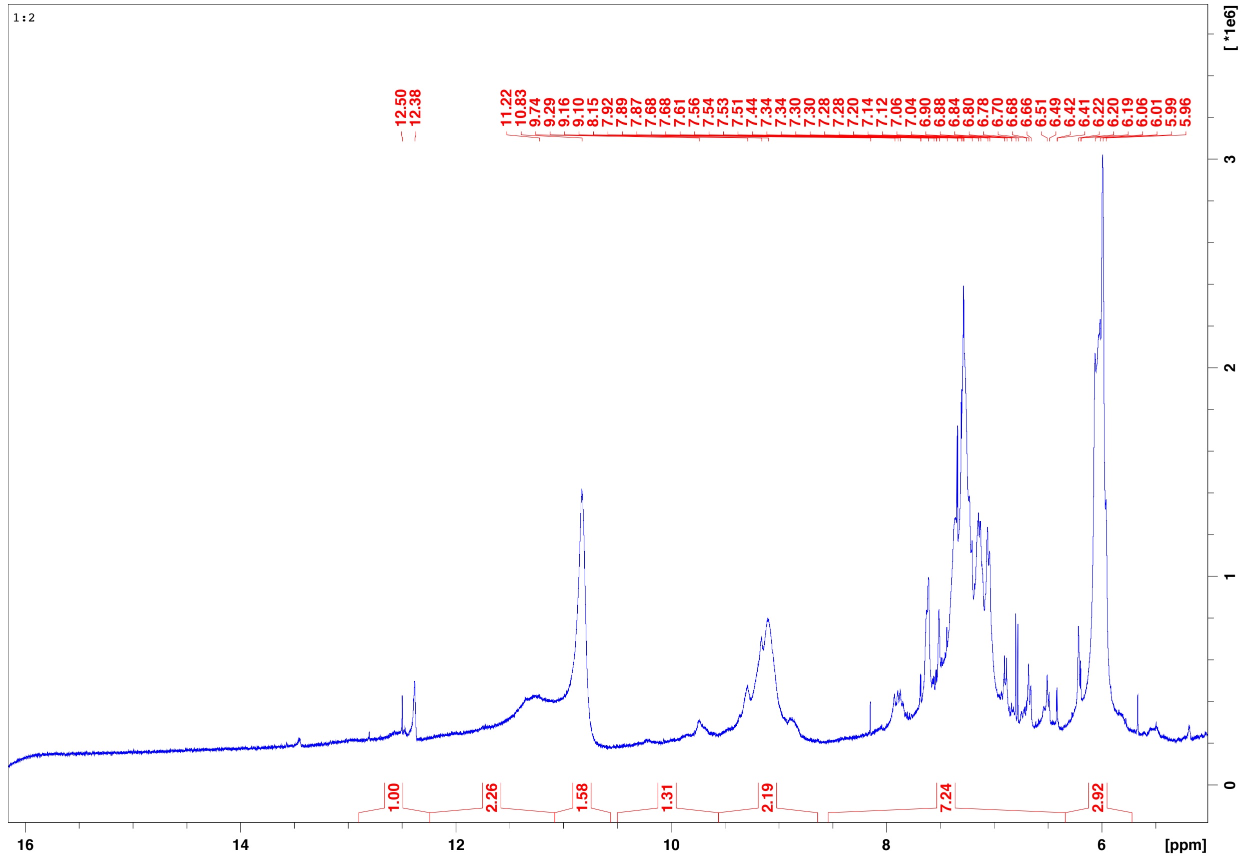

Figure S5. ^{1}H-NMR spectrum of NP2 (400 MHz, DMSO-d6). δ: 5.75 – 6.40 (Ar-H, m, H-6, H-8), 6.99 – 7.30 (Ar-H, m, H-5′, H-6′, H-2′); 8.65 – 9.55 (–OH, m, C7-OH, C4′-OH, C3′-OH), 10.70 – 11.50 (–OH, m, C3-OH), 12.36 – 12.65 (–OH, m, C5-OH) ppm.

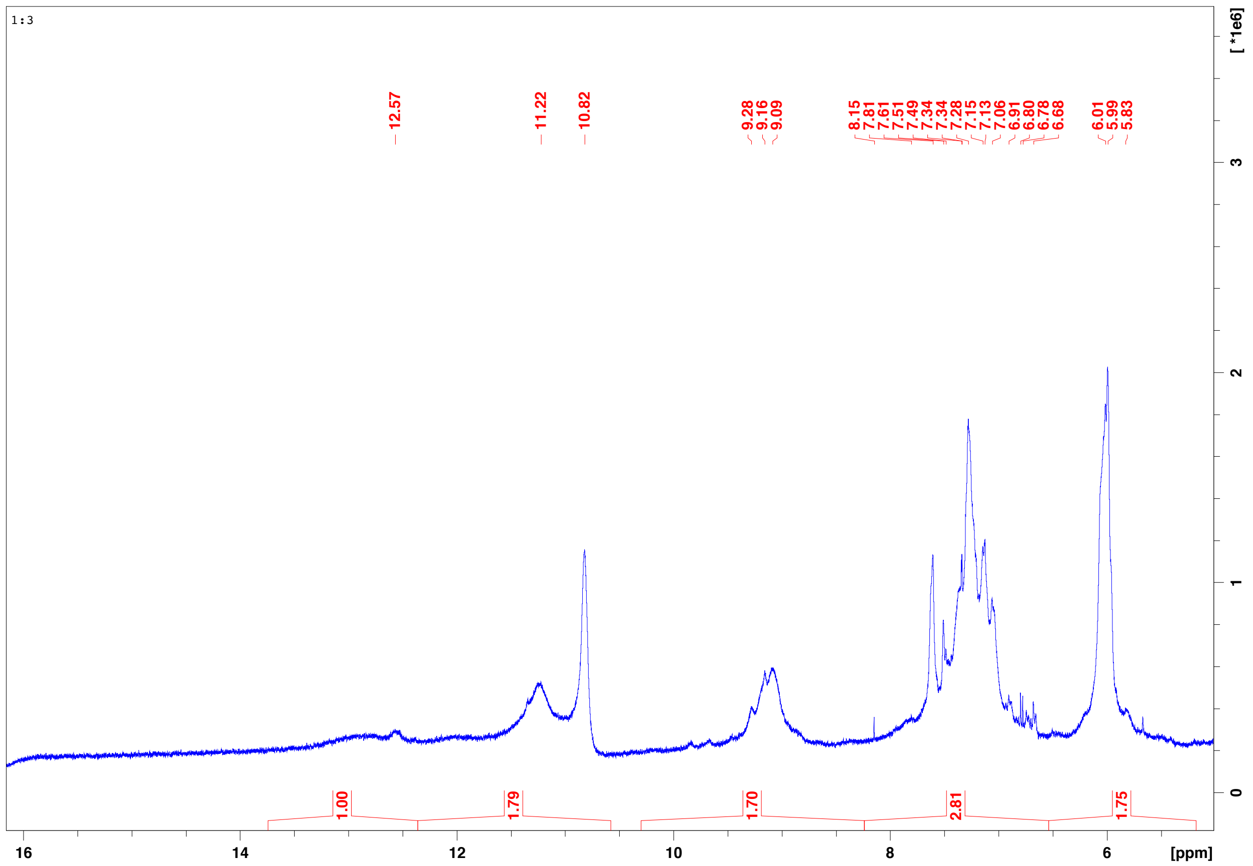

Figure S6. ^{1}H-NMR spectrum of NP3 (400 MHz, DMSO-d$_6$). δ: 5.99 (Ar-H, d, H-6, H-8), 6.90 – 7.70 (Ar-H, m, H-5′, H-6′, H-2′); 9.09 – 9.28 (–OH, m, C7-OH, C4′-OH, C3′-OH), 10.70 – 11.50 (–OH, m, C3-OH), 12.40 – 13.40 (–OH, m, C5-OH) ppm.

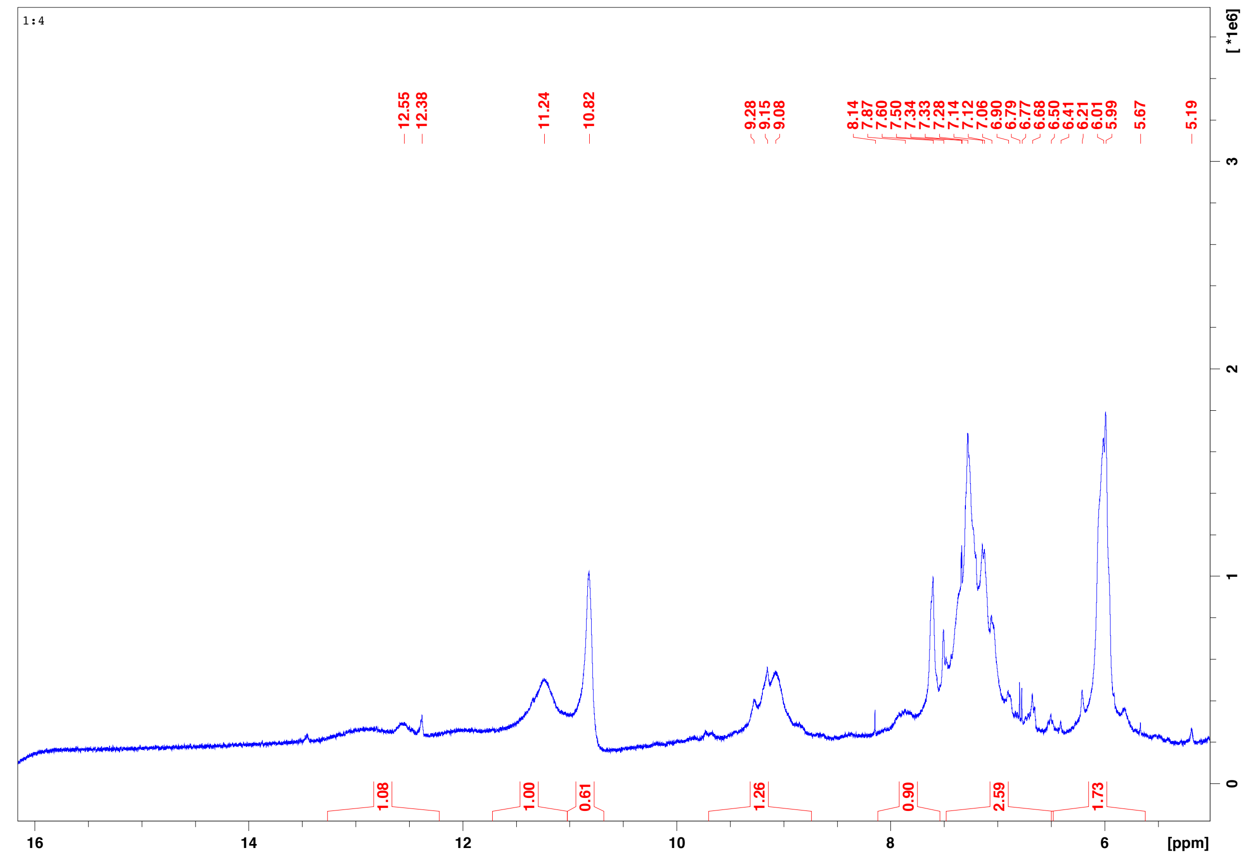

Figure S7. ^{1}H-NMR spectrum of NP4 (400 MHz, DMSO-d$_6$). δ: 5.99 (Ar-H, d, H-6, H-8), 6.80 – 7.75 (Ar-H, m, H-5′, H-6′, H-2′); 9.08 – 9.28 (–OH, m, C7-OH, C4′-OH, C3′-OH), 10.75 – 11.50 (–OH, m, C3-OH), 12.37 – 12.70 (–OH, m, C5-OH) ppm.

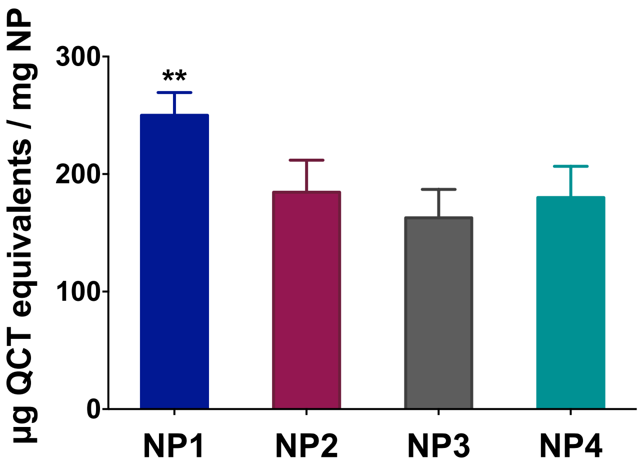

Figure S8. Total phenol content in QCT NPs expressed as µg QCT equivalents per mg NP.

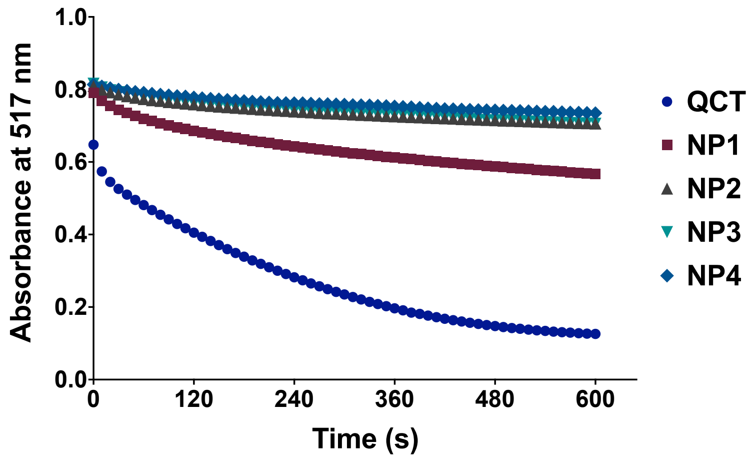

Figure S9. DPPH radical scavenging kinetics of QCT NPs compared to unmodified QCT over the first 10 min of the reaction. One hundred micrograms of each material was dissolved in 200 μL ethanol and added to 4 mL DPPH (0.1 mM in ethanol). The UV absorbance at 517 nm was immediately recorded at 10-second intervals up to 10 min.
